# All Photons Imaging Through Volumetric Scattering

**DOI:** 10.1038/srep33946

**Published:** 2016-09-29

**Authors:** Guy Satat, Barmak Heshmat, Dan Raviv, Ramesh Raskar

**Affiliations:** 1 Media Lab, Massachusetts Institute of Technology, Cambridge, MA 02139, USA

## Abstract

Imaging through thick highly scattering media (sample thickness ≫ mean free path) can realize broad applications in biomedical and industrial imaging as well as remote sensing. Here we propose a computational “All Photons Imaging” (API) framework that utilizes time-resolved measurement for imaging through thick volumetric scattering by using both early arrived (non-scattered) and diffused photons. As opposed to other methods which aim to lock on specific photons (coherent, ballistic, acoustically modulated, etc.), this framework aims to use all of the optical signal. Compared to conventional early photon measurements for imaging through a 15 *mm* tissue phantom, our method shows a two fold improvement in spatial resolution (4*db* increase in Peak SNR). This all optical, calibration-free framework enables widefield imaging through thick turbid media, and opens new avenues in non-invasive testing, analysis, and diagnosis.

Imaging through thick scattering media is a decades-old challenge in optics with numerous applications in biomedical and industrial settings. For a thin scattering barrier (<5 mm) the problem has been tackled by methods based on coherence^1^,^2^, time-reversal^3^,^4^,^5^,^6^,^7^, time-of-flight (ToF)^8^,^9^, and speckle correlations^10^,^11^. Thicker barriers require complex coupling between acoustical and optical elements (acousto-optic^12^ and photo-acoustic^13^,^14^) or measurement of intact ballistic photons^15^,^16^ which suffers severely from low signal-to-noise ratio (SNR). While ballistic photons carry superior image information quality^17^,^18^, the probability to measure them drops exponentially with the medium thickness^19^.

Conventional methods for imaging behind scattering barriers utilize a separating parameter (e.g. acoustic modulation^12^, time of arrival^15^,^20^, or coherence^1^,^2^) to lock into a distinct set of photons that have a contrasting character compared to the rest of the scattered light. While successful in some cases, this concept fundamentally rejects a large portion of the photons that could potentially contribute to signal recovery, especially in thick barrier scenarios where the majority of the signal is considered “scattered” and useless. The recent rise of computational imaging techniques and its alliance with ultrafast imaging^21^,^22^ have shown notable promise for extracting information^23^ from the wasted or ambiguous portion of the signal^24^. However they have not been demonstrated in remote sensing through volumetric thick scattering.

The use of diffused photons for imaging through thick barriers is known as Diffuse Optical Tomography (DOT)^25^,^26^,^27^, and was also demonstrated with a time-resolved measurement^28^. DOT has been used to image the human cortex^29^ and breast^30^. Unlike DOT, API illuminates the entire scene simultaneously and performs a dense measurement of the entire spatio-temporal response profile (which allows single shot measurement). Supplementary Note S1 and Supplementary Table S1 provide detailed comparison of API and DOT.

Another notable technique for laser imaging is LIDAR (Light Imaging Detection And Ranging). Unlike RADAR, which operates in longer wavelengths, LIDAR operates in the visible and near infrared spectrum and can suffer from diffusion and scattering. Overcoming this in LIDAR is known as Laser Imaging Through Obscurants (LITO)^31^, where the primary solution to overcome scattering is time gating^32^,^33^,^34^ which is equivalent to measurement of ballistic (unscattered) photons, and so it discards the majority of the incoming signal (diffused photons).

In this work, we develop “All Photons Imaging” (API) framework which revitalizes the conventionally lost portion of the signal, and demonstrate imaging through thick barriers. API is demonstrated here with time-resolved measurement and uses both early (ballistic and snake) and diffused photons. API is an all optical, calibration-free framework which enables widefield imaging through thick highly scattering turbid media. Since API does not rely on intrinsic properties of the optical signal (for example coherence or polarization) it can scale well to long range sensing. This makes it appealing for biomedical applications such as full organ imaging^35^ as well as remote sensing configurations such as imaging through fog and clouds^36^.

## Results

**Figure 1:**
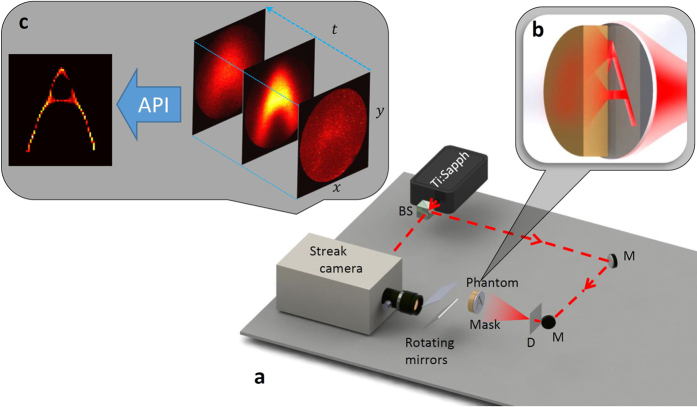
Imaging Through Thick Scattering. (**a**) Optical setup. A ti:sapphire laser is scattered by a diffuser sheet (D) to make a distant point source that is used to illuminate the mask. The mask is adjacent to the 15 *mm* tissue phantom which is imaged with a streak camera. (**b**) Schematic of the diffusion process inside the tissue phantom. (**c)** The optical setup captures the time dependent distorted signal of the mask. Each frame corresponds to a different arrival time of the distorted signal. Using API the mask is recovered.

Our optical setup arrangement is illustrated in Fig. 1. A pulsed remote point source back-illuminates a mask adjacent to a 15 *mm* thick tissue phantom. The sensor is a streak camera with a time resolution of 2 *ps* and a time window of 1 *ns* (see Methods). First, we construct a forward model for time-resolved volumetric light scattering, i.e. the space-time measurement *m*(*x, y, t*) as a function of the hidden scene s(x, y):


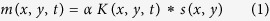


where *α* is an intensity scaling factor, K(x, y, t) is a scattering kernel and ‘*’ denotes convolution over (x, y). The kernel *K* blurs the signal in a time variant manner, such that the temporal information allows us to increase our measurement diversity (each frame is a different distorted measurement of the target) and recover the hidden scene.

The optical path of each photon is a realization of a random walk process. The forward model captures the mean of this random walk over a large number of photons. We consider the kernel *K*(*x*, *y*, *t*) as the probability density function of measuring a photon at position (*x*, *y*) and time *t*. Using this probabilistic formulation the kernel can be decomposed to:


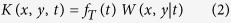


such that *f*_*T*_(*t*) is the probability density function of measuring a photon at time *t* (for example, this term captures the small probability of measuring a ballistic photon as opposed to a diffused photon), independent of its location, and *W*(*x*, *y*|*t*) is the probability density function of measuring the photon at position (*x*, *y*) given the time *t*. Since *W*(*x*, *y*|*t*) is a probability density function it should be normalized to 1, and therefore has a normalization factor that depends on *t*. For simplicity in our derivation, we absorb that factor in *f*_*T*_(*t*). *W*(*x*, *y*|*t*) is a time-dependent scattering kernel:


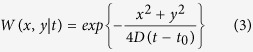


where, *D* is the diffusion coefficient, and *t*_0_ accounts for time shift due to the thickness of the sample. Our method is calibration-free, and estimates *f*_*T*_(*t*), *D* and *t*_0_ directly from the raw measurements as discussed below. It should be mentioned that since *f*_*T*_(*t*) is estimated from the measurement itself, it captures information about all photon transmission modalities (ballistic and diffused), independent of their statistical model.

**Figure 2:**
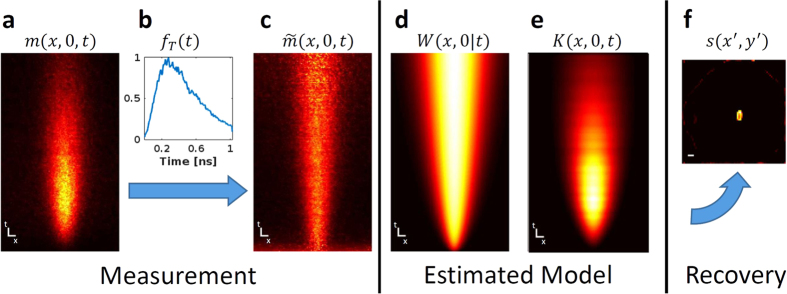
Forward Model for a 4 *mm* Point Source. (**a**) Raw measurement cross section (*x* − *t*) plane). (**b)** Recovery of the time probability function *f*_*T*_(*t*). (**c**) The normalized measurement cross section. (**d**) Result of estimating the model parameters *D*, *t*_*0*_. (**e**) Multiplying the time dependent diffusion kernel *W* by the probability function *f*_*T*_ results in a good match to the raw measurement, thus showing the suggested forward model captures the significant features of the signal. (**f**) The recovered scene using our method. Vertical scale bars equal 50 *ps*. Horizontal scale bar equals 5 *mm*.

We consider a point source of 4 *mm* hidden behind our 15 *mm* thick tissue phantom as a toy example to demonstrate the reconstruction procedure. A cross section of *m*(*x*, *y* = 0, *t*) is shown in Fig. 2a (we define (x, y) = (0, 0) as the center of the tissue phantom). The first step in our reconstruction flow is to estimate the probability function *f*_*T*_(*t*). We perform a search over (*x*, *y*) for the point in space (*x*_0_, *y*_0_) with the strongest signal and use it as *f*_*T*_(*t*) (Fig. 2b), i.e. *f*_*T*_(*t*) = *m*(*x*_0_, *y*_0_, *t*). We note that *f*_*T*_(*t*) doesn’t contain any spatial information and so it should not be a part of the reconstruction process. We normalize the measurement such that: 
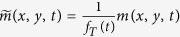
 (Fig. 2c). Next, we estimate *D*; we note that when comparing two frames from time points *t*_1_ and *t*_2_, we can write (assuming *t*_2_ > *t*_1_):


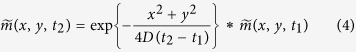


which is independent of *t*_0_. This allows us to perform a line search and fit *D* to the experimental measurement. Finally, in order to estimate *t*_0_ we search for the first frame with signal above the noise floor. The result is an empirical estimate for *W*(*x*, *y*, *t*) (Fig. 2d). The calculated *K*(*x*, *y*, *t*) captures the key features of the signal as depicted by its cross section in Fig. 2e.

In order to complete the inversion process we utilize *W*(*x*, *y*, *t*) as an empirical forward model, and compute the expected measurement 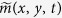 for a point source in any position on the hidden scene plane. These computed measurements are lexicographically ordered and stacked into a matrix ***A*** such that each column in ***A*** is the expected measurement for a specific point source location. Our goal is to calculate the hidden scene *s* from the normalized spatio-temporal measurement 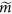 by solving 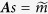, where ***A*** captures the forward model which is a mapping from spatial coordinates to spatio-temporal coordinates. Since ***A*** is a blurring operator (both in space and time) and thus non-invertible, we translate the inversion problem to an optimization framework and add an *l*_1_ regularizer:


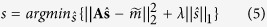


where *λ* is the regularization parameter, and 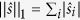. To solve the optimization problem we use fast iterative soft thresholding (FISTA)^37^. We initialize the algorithm with the noisy ballistic photon frame *m*(*x*, *y*, *t*_0_) to help the algorithm quickly converge to the solution (Fig. 2f). The inputs to the FISTA algorithm are the forward operator as defined by Eq. 3 (after evaluating the model parameters, i.e. the matrix ***A***), the normalized measurement 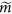 (i.e. the full normalized spatio-temporal profile), and an initialization image which we choose to be the first frame above the noise floor.

**Figure 3:**
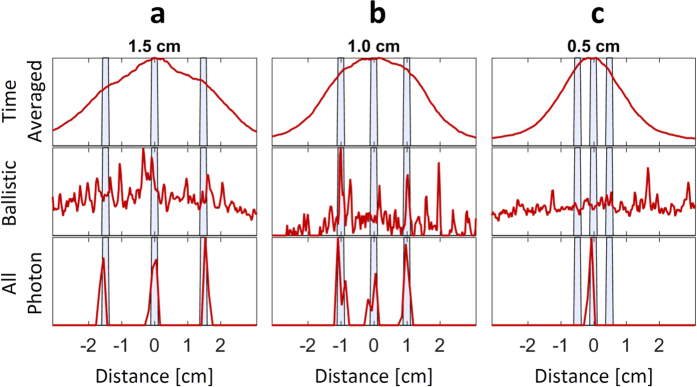
Recovery of Slits with API. Three slits separated by 15, 10 and 5 *mm* (**a**–**c**) respectively and their recovery with Time Averaging and Ballistic photons compared to API. Blue shadings are the slits′ location ground truth. Our method shows significant advantages in recovering the three slits when they are separated by up to 5 *mm*, while the other methods fail in all cases.

To evaluate our method we place masks composed of three slits separated by 15, 10 and 5 *mm* behind the thick diffuser (Fig. 3), and compare our results to a time-averaged measurement and a ballistic photons measurement. The former method, which integrates over the entire exposure time and does not use temporal information, results in a blurry image, as predicted. When utilizing ballistic photons^15^ the correct location of the slits cannot be recovered since the signal is comparable to the measurement noise. As opposed to these two methods which fail to find the correct locations of the three slits for the 15 and 10 *mm* cases, our method successfully recovers this information. For the 5 *mm* separation, however, our approach fails as it is below the recoverable resolution of our system (see further analysis below).

**Figure 4:**
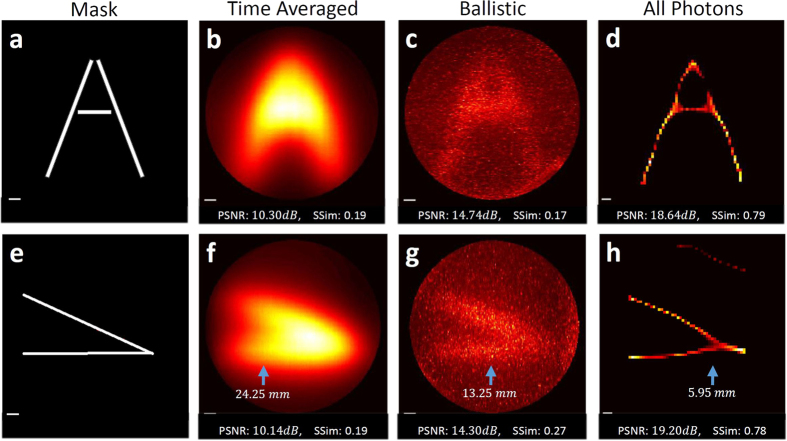
Recovered Scenes. (**a**) Hidden mask shaped like the letter ‘A’. (**b**) Recovered scene without using time-resolved data; the result is very blurry. (**c**) Recovered scene using only ballistic photons; the signal is embedded in the noise level. (**d**) Recovered scene using API; the result clearly recovers the hidden scene. (**e–h**) Mask and results for wedge-shaped scene. Blue arrows mark the points used to evaluate best recoverable resolution and the corresponding resolution. All reconstructions are quantitatively evaluated with both PSNR and SSim (ranges in [0, 1], higher is better). Scale bar equals 5 *mm*.

To demonstrate the recovery of two-dimensional scenes using our method, we place a few masks behind the thick diffuser and apply our reconstruction algorithm. First we use a mask of the letter ‘A’ (Fig. 4a). While the time averaging result is blurry and the information content of the scene is gone, the ballistic photons capture some of the information, but the signal is comparable to the measurement noise. However, our method is clearly able to capture the information content of the scene (Fig. 4d). Similarly, we place a wedge-shaped mask behind the diffuser and demonstrate reconstruction with the different techniques (Fig. 4e–h). The wedge shape provides the resolution limits of the different methods, marked by blue arrows, and the corresponding resolution in *mm* is overlaid on the reconstructions. Finally, to quantitatively evaluate our method, we calculate the peak signal to noise ratio (PSNR) and structural similarity index^38^ (SSim) of the different methods with respect to the baseline mask. While PSNR makes a point by point comparison, SSim takes into account the structure and spatial information of the images (SSim ranges in [0, 1], higher is better).

Both examples demonstrate the same trend in results. Time-averaging produces the worst results (though the image is noiseless, it is very blurry). Ballistic photons produce, as expected, a very noisy result. While we may observe some of the target features, they are comparable to the noise level, and contain some blur. Attempts to de-blur the ballistic photons results will fail due to significant noise. API is able to capture the information content of the scene, which is verified by the quantitative metrics. Supplement videos 1 and 2 show the recovery process of the algorithms for the two scenes. Supplementary Fig. S3 shows results for more complicated scenes.

## Discussion

**Figure 5:**
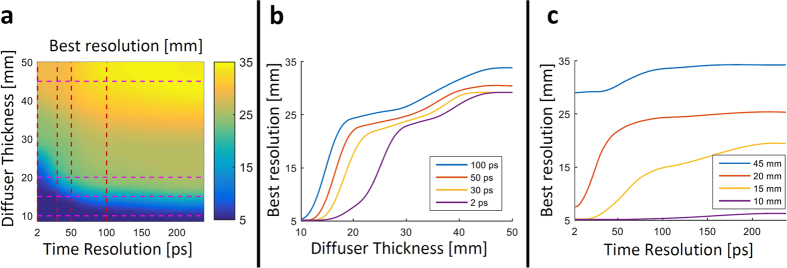
API Performance Analysis. (**a**) Monte Carlo simulation results for varying sensor time resolution and diffuser thickness; colors represent the best recoverable resolution. (**b**) Vertical cross sections (for different time resolutions). (**c**)Horizontal cross sections (for different diffuser thickness).

In order to analyze the correlation between the sensor time resolution and the diffuser parameters, we perform Monte Carlo simulations to simulate various diffuser thicknesses (the number of simulated photons is 10^9^, in a sample with scattering coefficient of 200 cm^−1^ and anisotropy coefficient of 0.85). These simulated measurements are then used in order to evaluate the best recoverable scene resolution as a function of the sensor temporal resolution and diffuser thickness. Fig. 5 presents these results. As predicted, better temporal resolution of the sensor enhances the measurement diversity and allows better resolution. To further demonstrate this trend we plot several cross sections of different sensor temporal resolutions (Fig. 5b), and several cross sections of different diffuser thicknesses (Fig. 5c). We note that for sensor time resolution below 50 *ps* we gain exponentially better scene resolution for increasing diffuser thickness. This is especially true thicknesses in the range of 12–30 *mm*. This shows the benefit of measuring with 2 *ps* time resolution, since a measurement with faster time resolution provides better recoverable resolution. Specifically, the improved time resolution inputs the reconstruction framework with a more diverse set of measurements, which increases the robustness of the inversion process and allows better recoverable resolution. For thinner diffusers better time resolution has little added value, and for thicker diffusers better time resolution improves the recovered resolution linearly. To better understand how the diffuser thickness and time resolution both affect the recoverable resolution, we consider the PSF presented in Fig. 2. As the diffuser gets thicker, the distribution along the *x* − y − *t* axes broadens. Improved time resolution allows capturing these changes more accurately, thus it provides a more diverse set of measurements and allows to recover better spatial resolution.

The number of photons that can arrive through 15 *mm* of tissue phantom for a single pulse is not sufficient to provide acceptable SNR for our algorithm. This forces integration over thousands to millions of pulses which corresponds to our 100 *ms* integration time. Though we did not seek to show the fastest integration time, it is certain that an increase in power level and repetition rate of the laser can reduce this integration time. Long integration can be a bottleneck for applications that require tracking of dynamic scenes such as those found in cytometry systems. To further study this possible limitation we performed a Monte Carlo simulation for the required PSNR for API. As seen in Supplementary Fig. S1, the PSNR of the system (which can increase with longer integration time, higher laser repetition rate or laser power) directly affects the reconstruction resolution. For example, the measurement PSNR in our system (61.7 *dB*) is well above any noise limitation of API (we notice performance degradation for PSNR below 39 *dB*), so the integration time can be shortened without impact on the reconstruction quality. Supplemental Fig. S3 shows successful reconstruction of targets with measurement PSNR below 45 *dB*.

The setup used in this study demonstrates the concept in transmission mode. This can be applied directly to applications such as mammography, where both sides of the tissue are accessible. However, the method can be extended to remote sensing and in reflection mode by several means that generate synchronized pulses in the medium, such as nonlinear effects, e.g. two-photon^39^ and localized plasma discharges^40^, which can be used for atmospheric studies^41^.

Using an *l*_1_ regularizer to invert Eq. 5 is common in inverse problems^42^. We note that any scene can be represented with a basis in which it will be sparse; specifically, we can write *s *= *Bx* where *B* is the basis and *x* is a sparse vector. Eq. 5 then becomes:


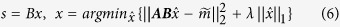


For example, natural scenes are known to be sparse in gradient domain. This problem has been well studied with available solutions like TWIST^43^ and TVAL3^44^. We note that the use of sparsity-based regularizers has been proven to be very beneficial for inverse problems in imaging^42^. However, there might be rare cases in which absolutely nothing is known about the target or its statistics. In that case, inversion can be performed with traditional Tikhonov based l2 regularizer or the Moore-Penrose general inverse^25^. While they require no target priors, the reconstruction result tends to be blurrier and with lower resolution. Comparison between reconstruction based on Eq. 5 and Moore-Penrose inverse are presented in Supplementary Fig. S4.

API is compatible with time-resolved measurement devices that require the same resources to measure ballistic and diffused photons; for example, a streak camera measures all photons within the same sensor swipe, and a single photon avalanche photodiode (SPAD) array will measure ballistic or diffused photons regardless of the SPAD parameters (only the source and geometry will change this probability). Thus, instead of relying only on ballistic photons, which would require extremely long integration times, API allows using the otherwise wasted photons and reduce the measurement time accordingly. API can also be compatible with LIDAR for remote sensing. As previously mentioned, the main solution to overcome scattering with LIDAR is time gating, which is similar to only measuring ballistic photons (non-scattered photons in the context of reflection mode). API can improve this setting by using the scattered photons as part of the signal used for reconstruction. Since many LIDAR systems are pulse based, the time-resolved measurement used for basic LIDAR can provide the required measurement diversity for API (if the gate time is short enough according to the analysis provided in Fig. 5).

This study considered only homogeneous scattering volumes. However, we note that API can scale to piecewise smooth volumes with variations along the *z* axis. This is demonstrated in Supplementary Fig. S2, which shows simulation results for a scattering medium composed of two layers (each layer is 7.5 *mm* thick). We varied the scattering coefficient of the two layers, and for each configuration performed a Monte Carlo simulation and estimated the recoverable resolution with API. The results show no dependency on the two individual scattering coefficients which are chosen in the range: *μ*_*T*_ ∈ [100, 300]. This shows that API is applicable for imaging through layered structured mediums (e.g. skin tissue). Variations in the (*x*, *y*) axes are more complicated; in such cases variations of low amplitude will be absorbed by the current robustness of API (see Supplementary Fig. S1 for discussion on API’s noise sensitivity). Stronger variations will cause significant model mismatch that requires iterating between estimation of the spatially dependent blur operator *K*(*x*, *y*, *t*:*x*′, *y*′) and the target scene *s*(*x*′, *y*′). This will be a topic of future research.

In summary, we present a robust, calibration-free and widefield method to recover scenes hidden behind thick volumetric scattering media. As opposed to conventional, all-optical methods, which require locking on a specific part of the optical signal, our method uses all photons and depends on measurement diversity to recover the hidden scene. This results in better SNR performance and improved resolution. We show that the measurement diversity gained by exploiting time dependency allows novel computational techniques to improve the overall system performance. Future work might include other physical parameters to increase measurement diversity. Our computational framework can also be used with other time-resolved sensors, such as SPAD arrays, and could also be combined with coherent-based optical methods. Our results pave the way for integration of pure physics-based methods with novel computational frameworks.

## Methods

### Optical Setup

A Ti:Sapph (795 *nm*, 0.4*W*, 50*fs* pulse duration, 80 *MHz* repetition rate) is focused onto a polycarbonate thin diffuser (Edmund Optics, 55–444) 40 *cm* away from the sample, and illuminates the Intralipid tissue phantom (reduced scattering coefficient of 10 *cm*^−1^) from the back (Fig. 1). The sensor is a streak camera (Hamamatsu C5680) with a nominal time resolution of 2 *ps* and a time window of 1 *ns*. The sensor has a 1D aperture and records the time profile of a horizontal slice of the scene (*x* − *t*). A set of two motorized mirrors is scanning the *y* axis of the scene in a periscope configuration to measure a 305 × 305 × 512 tensor for *x*, *y*, *t* axis respectively, where each entry corresponds to 0.3 *mm* × 0.3 *mm* × 2*ps*. The exposure time of each *x* − *t* slice is 100 *ms* (total acquisition time is 30 *sec* per scene). The problem of measuring the full *x* − *y* − *t* cube in a single shot has already been solved by e.g. time-space multiplexing^45^,^46^ and compressive techniques^47^,^48^. We plan to integrate these methods into our optical setup in a future study.

### Algorithm Parameters

The recovered scenes have a resolution of 70 × 70 pixels, where each pixel corresponds to 1 *mm*. The optimization problem in Eq. 5 is solved with Fast Iterative Soft Thresholding (FISTA)^37^, with 10000 iterations. The regularization parameter is set to *λ* = 0.004 for all scenes. The algorithm’s run time is approximately 1 minute with unoptimized Matlab code on a standard desktop computer with full data resolution.

## Additional Information

**How to cite this article**: Satat, G. *et al*. All Photons Imaging Through Volumetric Scattering. *Sci. Rep.* **6**, 33946; doi: 10.1038/srep33946 (2016).

## Supplementary Material

Supplementary Information

Supplementary Video 1

Supplementary Video 2
