# Supplementary material for: All Photons Imaging Through Volumetric Scattering
Source: Sci Rep. 2016 Sep 29;6:33946. doi: 10.1038/srep33946 (PMC5041145; doi:10.1038/srep33946)
Supplement: Supplementary Information [file srep33946-s1.pdf]

All Photons Imaging Through Volumetric Scattering Supplementary Material

Guy Satat*[1], Barmak Heshmat[1], Dan Raviv[1], Ramesh Raskar[1]

[1]*Media Lab, Massachusetts Institute of Technology, Cambridge, MA 02139, USA*
*guysatat@mit.edu

Supplementary Videos

The videos present the iteration process of the FISTA algorithm, starting with the initialization image, followed by the intermediate result of each iteration (estimated target). The frame-rate of the movie changes, so that it's slower during the first iterations while there are significant changes from iteration to iteration, and faster later when there is less change. The videos show how the iterative reconstruction process converge to the correct solution.

Video S1: Algorithm iteration process for 'A' shape scene. The video shows the convergence of the reconstruction algorithm to the correct solution.

Video S2: Same as Video 1 for wedge shaped scene.

Supplementary Figure S1 - API PSNR sensitivity analysis

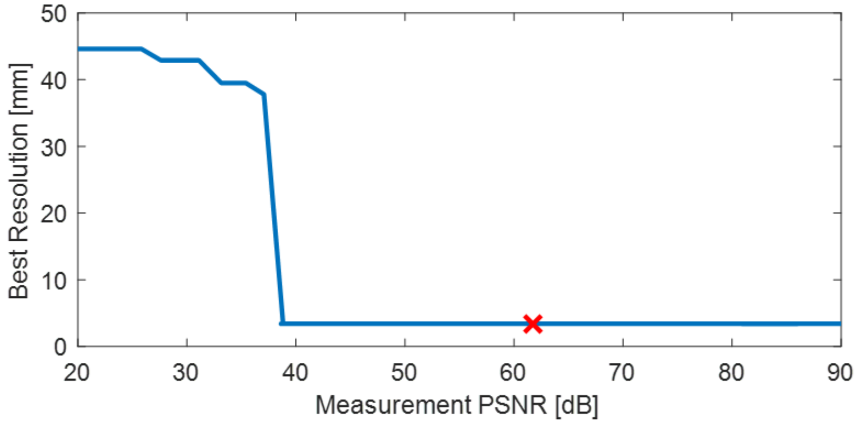

Measurement PSNR effect on recoverable resolution is evaluated by adding noise to the measurement and performing API. We perform a Monte Carlo simulation with 10^9 photons passing through a 15 mm thick phantom with extinction coefficient of 200 cm^{-1} and anisotropy coefficient of 0.85. We then add white Gaussian noise to the measurement and perform API to evaluate the recoverable resolution. We note that API handles PSNR above 39 dB very robustly. For lower PSNR the first step of API (estimation of model parameters) fails, which results in a significant loss of performance. We also evaluate the measurement PSNR of our system by using the method suggested by Xinhao[1] and find it to be 61.7 dB, well within the stable behavior of API. This hints that the acquisition time of our system could have been significantly shorter without loss of performance (see also Supplemental Fig. S3).

Supplementary Figure S2 - API with inhomogeneous medium

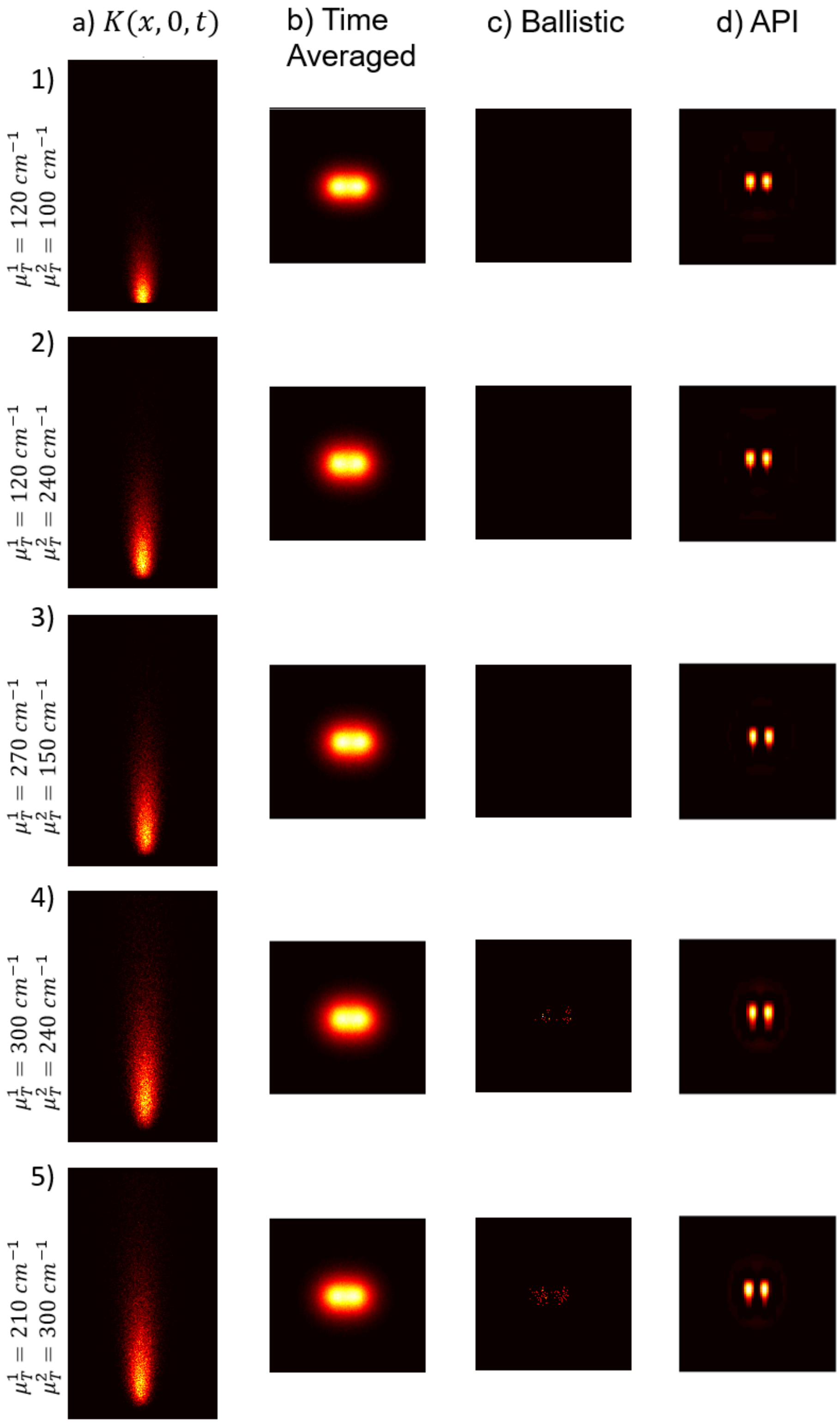

API with layered inhomogeneous medium. We perform a Monte Carlo simulation of 10^7 photons passing through 2 layers of scattering medium with scattering coefficients of μ_T^1, μ_T^2 respectively. Each layer is $7.5\ mm$ thick such that the total structure is $1.5\ cm$ thick. We find that for $\mu_T^1, \mu_T^2 \in [100,300]$ the recoverable resolution is $5.2\ mm$, i.e. it is independent of the independent values. The figure shows several examples of μ_T^1, μ_T^2 (rows 1-5). a) An $x - t$ cross section of $K(x, y, t)$ which shows the subtle changes in the PSF due to variations in the scattering coefficients. b) Failed recovery of the points without time-resolved data. c) Recovery of the points only with ballistic photons; note that this is a noiseless simulation, and any added noise would significantly impact the ability to recover the two sources with this result (see more examples in Supplementary Fig. S3). d) Successful recovery of two points $5.2\ mm$ apart with API, independent of scattering coefficients variation.

Supplementary Figure S3 – Additional reconstruction results with measurement noise below 45 dB

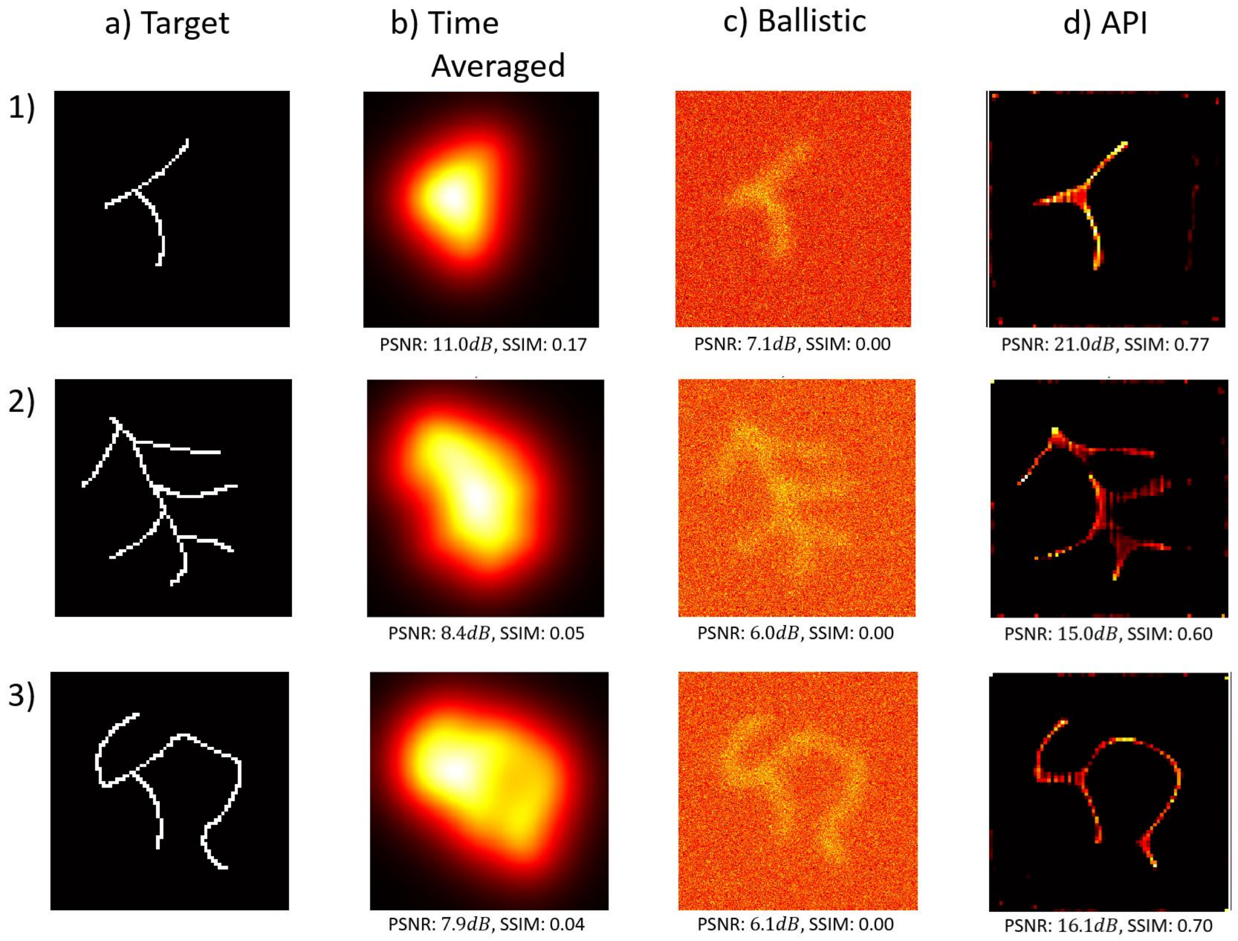

API successfully reconstructs a blood vessel-like structure with added measurement noise. The forward model derived from the experimental measurements is used to simulate the time-resolved measurements of targets 1-3, with added measurement noise that correspond to PSNR of: 42.5 dB, 44.5 dB, 43.9 dB respectively. a) The target. b) Recovery without time-resolved data. c) Recovery only with ballistic photons. d) Successful recovery with API. Below each reconstruction are the quantitative reconstruction metrics of PSNR and SSIM (ranges in [0,1], higher is better).

Supplementary Figure S4 – Comparison between FISTA and Moore-Penrose inversion

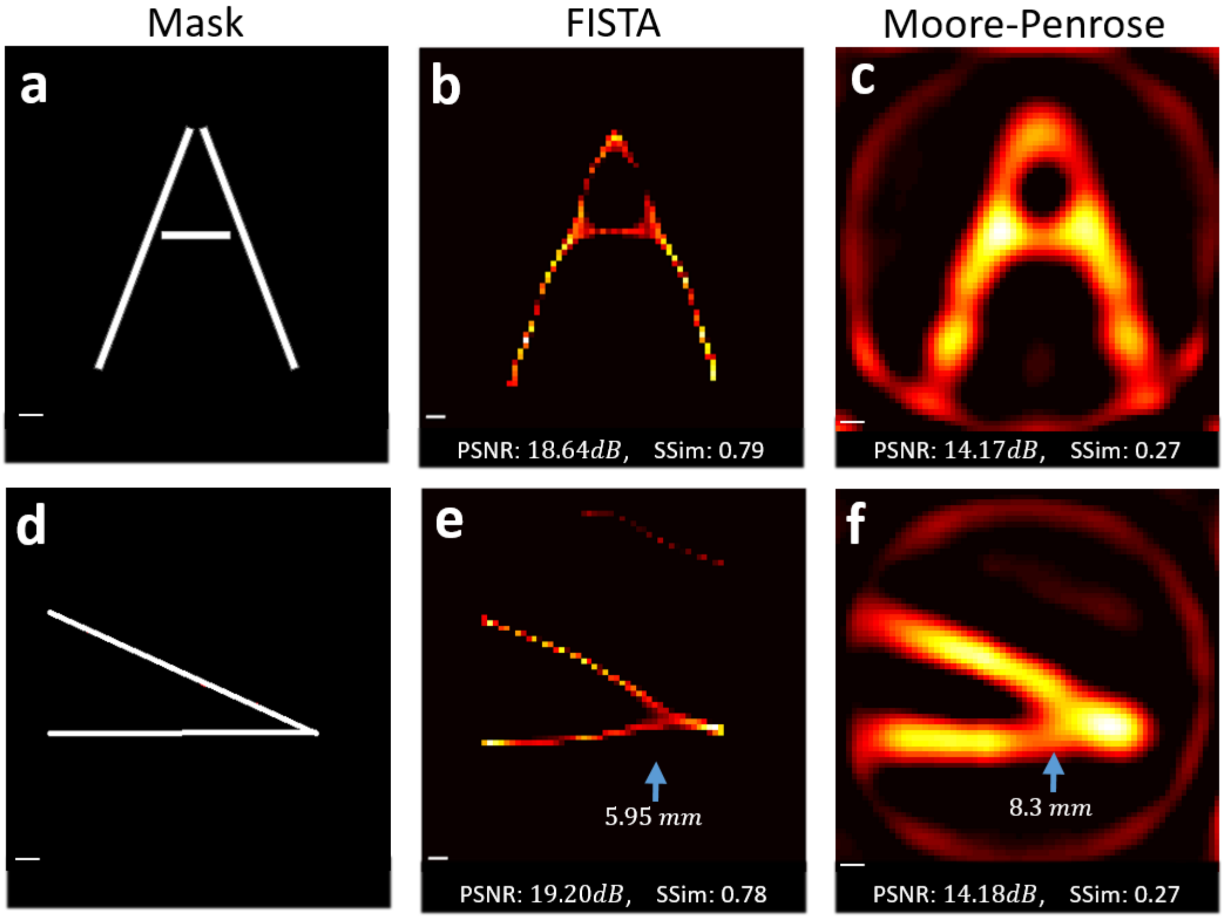

We compare the reconstruction result of a FISTA based inversion (with Eq. 5 from the manuscript, and Fig. 4 from the manuscript), and a Moore-Penrose based inversion defined by: $x = (A^T A + \alpha I)^{-1} A^T b$, with $\alpha = 10^{-4}$. We note that the Moore-Penrose based inversion result is blurrier and, as a result, with poorer recoverable resolution. The figure compares inversion results for the masks presented in Fig. 4 of the manuscript. (a) The target mask of letter 'A'. (b) Reconstruction result with FISTA. (c) Reconstruction result with Moore-Penrose inverse. (d)-(f) similar for wedge-shaped mask. Blue arrows mark the points used to evaluate best recoverable resolution and the corresponding resolution. The reconstructions are quantitatively evaluated with both PSNR and SSim (ranges in [0,1], higher is better). Scale bar equals 5 mm.

Supplementary Note S1 – Comparison between API and DOT

API is compared to DOT in Supplementary Table S1. We note that unlike DOT, API is based on a dense measurement of the entire spatio-temporal scene response, which allows robust inversion. API provides flexible wide field of view (unlike DOT which is usually based on a rigid structure with fixed optics). DOT usually requires raster scanning of the illumination source (or sequential illumination of multiple sources), while API enables a single shot measurement of the entire field of view (we note that this capability is based on coupling API with single shot measurement techniques in a streak camera as discussed in the Methods section).

Supplementary Table S1 - Comparison of API to DOT

	API	DOT
Measurement and use of full-dense spatio-temporal profile	Yes	No, DOT performs a sparse sampling of space. Some methods use full field cameras (but even if they perform time resolved measurement, it is of low time resolution).
Entire scene illuminated simultaneously	Yes	No, the illumination source is raster scanned or multiple sources are illuminated sequentially.
Requirement for raster scanning	Implementation with Scanning. Single shot is also possible[2,3].	Either sequential illumination (with multiple sources) or raster scan of single source.
Field of view	Variable – Illumination is flood illumination so it doesn't pose a restriction. Measurement is done with a remote camera, so wider/smaller field of view is a simple function of camera lens.	Fixed, usually based on rigid systems. Some methods use a standoff camera but still require raster scanning (i.e. limited flexibility in illumination field of view).
Contact with target	No contact (applicable to remote sensing).	Usually requires contact.
Imaging inside deep tissue	Yes	Yes

Bibliography

1. Liu, X., Tanaka, M. & Okutomi, M. Single-image noise level estimation for blind denoising. *IEEE transactions on image processing* **22,** 5226–37 (2013).

2. Liang, J., Gao, L., Hai, P., Li, C. & Wang, L. V. Encrypted Three-dimensional Dynamic Imaging using Snapshot Time-of-flight Compressed Ultrafast Photography. *Scientific reports* **5,** 15504 (2015).

3. Heshmat, B., Satat, G., Barsi, C. & Raskar, R. Single-shot ultrafast imaging using parallax-free alignment with a tilted lenslet array. *Cleo: 2014* **1,** STu3E.7 (2014).
